# Supplementary material for: Relationship between Psoriasis and Prevalence of Cardiovascular Disease in 88 Japanese Patients
Source: J Clin Med. 2021 Aug 17;10(16):3640. doi: 10.3390/jcm10163640 (PMC8397130; doi:10.3390/jcm10163640)
Supplement: Supplementary file 1 [file jcm-10-03640-s001.zip › jcm-1289127-supplementary.pdf]

Supplementary Materials

Table S1. Characteristics of patients with psoriasis at Kansai Medical University in detail.

Case	Age	Sex	Type	PASI	BMI	CCTA	Complications	Additional Treatment	CRP	BP	FHP	FHC	Hyperlipidemia	Diabetes	Smoking	Drinking	ABI
1	64	F	PsA	8	21.84	-	-	-	0.133	104/69	-	+	-	-	-	+	2
2	44	F	PsA	13	27.55	-	Cerebral infarction	-	0.08	144/100	-	-	+	-	+	-	-
3	72	M	EP	22	26.18	+	-	-	0.025	106/75	-	-	+	+	+	+	4
4	46	F	PsA	12	28.52	-	-	-	0.319	144/95	-	+	+	+	+	+	-
5	56	M	PV	16	28.13	-	-	-	0.073	127/96	-	-	+	-	-	+	-
6	57	M	GPP	8.9	22.6	-	-	-	0.077	137/87	-	+	+	-	+	+	-
7	53	M	PV	21	21.89	+	-	medication	0.09	151/97	-	-	-	-	-	+	-
8	81	M	PsA	11.2	24.61	+	Thoracic aortic aneurysm	madication	0.03	160/111	-	-	-	-	-	-	-
9	60	M	PsA	7.1	21.97	+	-	madication	0.05	131/94	-	-	-	-	-	+	-
10	64	M	PV	7.4	25.59	-	-	-	0.041	134/85	-	-	+	-	+	+	-
11	54	F	PV	5	28.04	+	-	-	0.03	153/93	-	-	-	-	-	+	-
12	48	M	PsA	10	22.79	-	-	-	0.162	146/113	-	+	-	-	-	+	-
13	49	M	PV	20	32.65	-	Liver cirrhosis	-	0.054	139/94	-	-	-	-	-	-	-
14	72	M	PV	28	25.82	+	Colon polyp	CABG	0.045	162/80	-	-	+	+	-	-	-
15	76	M	PsA	12.9	21.23	+	Thoracic aortic aneurysm	operation	0.041	156/105	-	+	-	+	-	-	4
16	67	M	PV	2.6	22.66	-	-	-	0.846	137/97	-	-	-	-	-	-	-
17	72	F	PV	9.6	27.34	-	-	-	0.105	156/84	-	+	-	-	-	-	-
18	42	F	PsA	16.2	21.91	-	-	-	0.078	116/78	-	-	-	-	-	-	-
19	71	F	PV	11.2	31.23	-	Angina	-	2.567	142/86	-	-	+	+	-	-	-
20	69	M	PV	29.3	23.03	-	-	-	0.059	142/99	-	-	+	-	+	+	-
21	79	M	PV	28	20.76	+	Tuberculosis	SMPI	0.315	154/103	-	-	-	-	-	+	-
**22	62	M	PsA	8	22.79	+	Cerebral hemorrhage	PCI	0.065	158/105	-	-	+	-	+	-	4
23	42	M	PV	33.9	37.18	-	-	-	0.41	150/82	-	-	+	-	-	-	-
24	41	M	PsA	6	21.8	-	-	-	2.377	124/84	-	-	-	-	-	-	3
25	35	M	PV	35	30.29	-	-	-	0.169	115/81	+	-	+	-	+	-	-
26	76	M	PsA	10	20.76	-	-	-	0.138	113/56	-	-	+	-	-	+	2
27	38	M	PsA	26.1	20.28	+	-	-	0.04	133/90	+	-	+	-	-	-	-

28	55	M	PsA	15.5	25.51	-	-	-	0.052	131/81	-	-	-	+	-	-	-
29	79	M	GPP	4	23.46	+	-	-	0.05	140/80	-	-	+	-	-	-	-
10	65	M	PV	10.6	24.22	+	-	madication	0.397	122/82	-	-	-	-	-	+	-
31	63	M	PV	15.9	26.86	-	-	-	0.184	139/67	-	-	+	+	+	-	-
32	33	M	PV	22	26.86	-		-	0.072	123/69	-	-	+	-	+	-	-
33	42	M	PV	14	22.2	-	-	-	1.678	120/78	-	+	-	-	-	-	-
34	83	M	GPP	30	28.2	+	Cerebral hemorrhage	SMPI	7.216	109/64	-	-	-	-	-	+	-
35	41	F	PV	8	19.92	-	-	-	0.037	103/50	-	+	-	-	-	-	-
36	41	M	PV	30	30.69	-	-	-	0.221	129/70	-	-	-	-	-	+	-
37	71	F	GPP	36	22.2	+	-	SMPI	0.075	167/95	+	-	-	-	+	+	-
38	56	M	PsA	4.5	29.07	-	-	-	0.285	120/81	-	+	-	-	-	-	-
39	64	M	PsA	2.9	24.31	+	-	madication	0.015	147/102	-	-	+	-	-	+	4
40	37	M	PV	6	25.35	-	-	-	0.344	127/80	-	-	-	-	+	-	3
41	74	F	PV	4	21.09	-	-	-	0.158	132/83	-	+	-	-	+	-	-
42	57	F	PsA	6	19.72	+	-	-	0.048	132/67	-	-	+	+	+	+	3
43	80	F	PsA	17.4	22.22	+	Angina	PCI	0.161	133/96	-	-	+	-	-	-	-
44	64	M	PV	10	22.68	-	-	-	0.037	115/76	-	-	-	-	-	+	-
45	67	F	PsA	3.1	21.37	-	Malignant lynphoma	-	0.028	136/88	-	-	+	-	+	+	-
46	45	M	PV	21.4	21.19	-	-	-	0.136	113/69	-	-	-	-	+	-	-
47	49	M	PV	20	22.76	-	-	-	0.053	120/70	-	-	-	-	-	-	-
48	51	M	EP	18.5	18.87	+	Lung cancer	-	0.051	105/62	-	-	+	-	+	+	-
49	79	M	PsA	2	20.08	+	-	SMPI	0.017	120/67	-	-	-	-	+	+	2
50	69	F	PsA	6	19.18	+	-	SMPI	0.138	167/60	-	-	+	+	-	-	4
51	70	F	PV	3	22.52	-	-	-	0.036	158/86	-	+	+	-	+	-	2
52	70	M	PsA	4	23.21	+	Myathenia gravis	SMPI	0.06	177/91	-	-	-	-	-	-	-
53	70	M	PsA	9.6	27.36	+	-	SMPI	0.197	177/104	-	-	+	-	-	-	4
54	48	M	PsA	2.8	22.22	-	-	-	0.028	115/73	-	+	-	-	+	+	2
55	58	M	EP	20	24.8	+	-	madication	0.034	138/98	-	-	+	-	+	+	-
56	54	M	PV	5.3	25.39	+	Heart failure	PCI、CABG	0.341	117/42	-	-	+	+	-	+	5
57	54	F	GPP	10.3	23.23	-	-	-	0.466	165/61	-	-	+	-	-	+	4
58	71	M	PV	4.8	24.01	-	-	-	0.053	139/82	-	-	+	-	-	+	4
59	47	M	PV	13	26.89	-	-	-	0.17	129/78	-	-	+	-	+	+	-
60	69	M	PsA	14.1	31.74	+	-	-	0.587	130/73	-	-	+	-	-	+	1
61	56	F	PV	3.9	27.77	-	-	-	0.041	134/86	-	-	+	-	-	-	2

62	68	M	PsA	5.4	29.18	+	-	-	0.062	147/84	-	-	+	-	+	-	4
63	49	F	PV	6	28.51	-	-	-	0.328	129/79	-	+	+	-	-	-	4
64	65	M	PV	10	26.89	+	-	SMPI	1.328	130/70	-	-	+	-	+	-	4
65	78	F	PsA	4	20	+	-	SMPI	1.095	124/73	-	-	-	-	-	-	3
66	36	M	PsA	4	30.12	-	-	-	0.201	109/65	-	-	-	-	-	-	2
67	69	M	PV	10	22.04	-	Angina	-	0.023	144/96	-	-	-	-	-	-	4
68	51	M	PV	13.2	20.61	-	-	-	0.193	117/79	-	-	-	-	+	-	4
69	39	M	PsA	4	31.71	+	-	SMPI	0.256	124/76	-	-	-	-	+	+	2
70	71	F	PV	10	18.08	-	-	-	0.032	147/72	-	-	-	-	-	-	2
71	38	M	EP	53.4	43.28	-	-	-	1.415	121/69	-	-	+	-	-	-	5
72	42	M	PV	1.2	31.07	-	-	-	0.654	155/103	-	-	+	-	-	+	-
73	68	M	PV	10	23.8	-	Pulmonary fibrosis	-	0.511	134/82	-	-	+	+	-	+	-
74	79	M	PsA	5.6	24.1	+	Liver cancer	-	0.347	154/79	-	-	+	+	-	+	4
75	56	M	PV	5	26.35	+	-	-	0.029	179/119	-	-	+	-	+	+	4
76	50	F	PsA	3.2	28.81	+	Uterine cancer		0.026	114/83	-	-	-	+	-	-	4
77	83	M	PV	4	23.5	-	Prostate cancer	-	0.053	139/89	-	-	-	+	-	-	2
78	74	F	PV	4.5	23.47	+	-	-	0.041	136/86	-	-	+	-	-	+	4
79	65	M	PV	4.9	25.22	-	Schizophrenia	-	0.235	132/83	-	-	-	-	+	-	2
80	55	F	PV	12.2	28.37	-	-	-	0.162	143/85	-	-	-	-	+	-	2
81	52	F	PV	5.4	25.53	-	Depression	-	0.115	138/99	-	-	+	-	-	-	2
82	48	M	PV	9.2	25.59	+	-	-	0.118	150/83	-	-	+	-	+	+	4
83	82	M	PV	4.8	31.83	+	-	-	0.086	156/89	-	-	-	-	+	+	2
84	42	F	PV	11.7	25.97	-	-	-	0.066	112/61	-	-	+	-	+	-	2
85	63	F	PsA	5.4	17.58	+	Renal failure	SMPI	0.491	129/61	-	-	-	-	-	-	4
86	68	M	PsA	2.4	24.03	+	Pulmonary fibrosis	CABG	0.113	131/79	-	-	-	-	-	-	4
*87	57	M	GPP	40	29.57	+	Angina	PCI	0.098	145/95	-	-	+	-	-	-	3
***88	69	M	PsA	2	22.15	+	-	-	0.103	133/93	-	-	+	-	+	+	2

＋: positive data, -: negative data, *: Case1, **: Case2, ***: Case3, ABI: ankle–brachial pressure index: Supple (1), Normal (2), Slightly stiff (3), Hard (4), Clogged (5), BMI: body mass index, BP: blood pressure, CCTA: coronary artery and cardiac computed tomography, CRP: C-reactive protein, DBP: diastolic blood pressure, EP: erythrodermic psoriasis, FHC: familial history of cardiovascular disease, FHP: familial history of psoriasis, GPP: generalized pustular psoriasis, PASI: psoriasis area and severity index, PsA: psoriasis arthritis, PV: psoriasis vulgaris, SBP: systolic blood pressure, TG: triglycerides.

Cases

Figures S1–S3

A subset of cases in which CCTA was performed is described as supplementary data (Figures S1–S3).

Case 1:

A 57-year-old man had been diagnosed with PV 20 years earlier, and had been receiving topical treatment, but PV was gradually worsening. About 5 years before this presentation, joint pain developed in all four limbs. Although he started treatment with nonsteroidal anti-inflammatory drugs, pain continued to exacerbate. A few days prior to presentation, he experienced chest pain and treatment (Percutaneous Coronary Intervention) for acute myocardial infarction was initiated. Subsequently, the patient visited the dermatology department for treatment of the skin eruptions. At this visit, palpable erythema was observed over virtually the whole body, including the head and neck area, and multiple pustules were present on the plaques. Joint pain was also identified in all four limbs (Figure S1a, b). PASI score was 40, BMI was 29.57 kg/m2, and blood pressure was 145/95 mmHg, indicating hypertension. Blood test results were normal, except for triglycerides (TG), which were high at 218 mg/dl (normal range: 29–155 mg/dl). CCTA revealed catheter placement in the left coronary artery (Figure S1c).

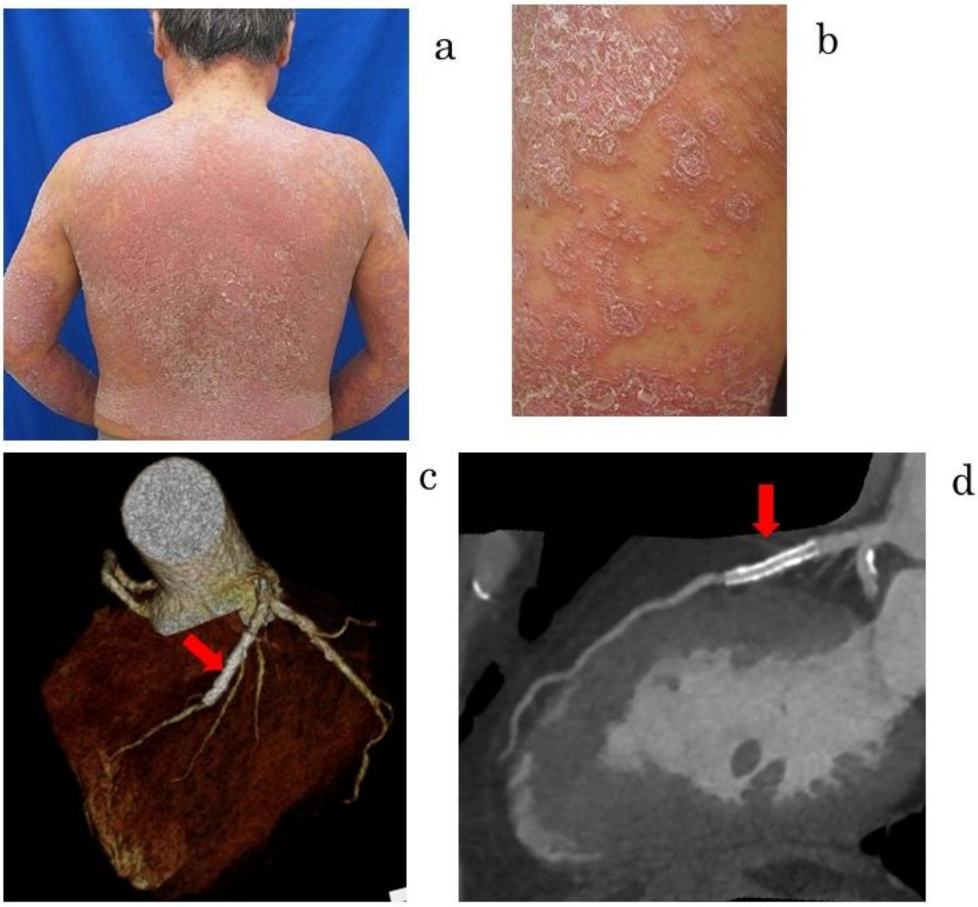

Figure S1. Severe case of psoriasis, Figure S1a: Almost the entire body surface displays irregular erythema, with pustules concentrated in areas of erythema. The patient has pain in the joints of all limbs, and PASI score is 40. **Figure S1b:** Magnification: pustules have formed and fused with scaly erythema. **Figure S1c, d:** c: CCTA, volume-rendering [VR] image; right: curved plane reconstruction [CPR] image). d: Stent placement (red arrow) is confirmed in the proximal part of the left anterior descending coronary artery. No stenosis is evident within the stent.

Case2:

A 62-year-old man without significant medical history had been diagnosed with PV 6–7 years earlier and was treated primarily with ultraviolet therapy, etretinate, and topical treatment by a local

physician. However, since skin eruptions had recently worsened, the patient visited our hospital and requested treatment with biologic agents.

On that visit, scaly erythema was observed on the head, back of the hands, and around the nail plates, and the nail plates were deformed (Figure S2a, b). Moreover, thick, scaly erythema was observed on the elbow and buttocks (Figure S1c, d). PASI score was 8, BMI was 22.79 kg/m2, and blood pressure was high, at 158/105 mmHg. Aside from mildly elevated TG (156 mg/dl; normal, 29–155 mg/dl), no abnormalities were observed on blood testing. MRI of the brain showed a hypertensive hemorrhage scar. Since CCTA revealed significant stenosis (90%) in the left descending and circumflex branches (Figure S2c), percutaneous catheter dilatation of the coronary artery was performed.

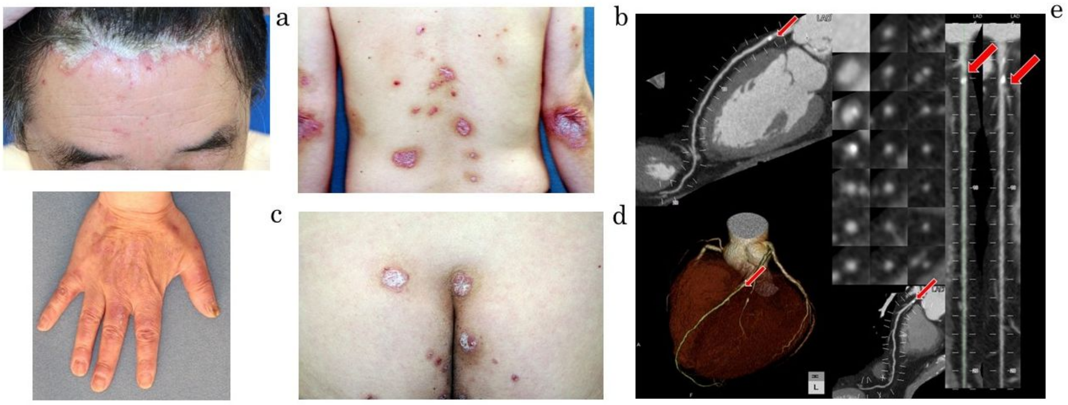

Figure S2. M**oderate case of psoriasis, Figure S2a–d:** Erythema with scales is apparent on the head (**a**), fingers and backs of the hands (**b**), backs of the elbows (**c**) and buttocks (**d**). The nail plates are deformed, and the patient has pain in the wrist and finger joints. PASI score is 8. **Figure S2e:** CCTA. Left: VR image; right: CPR image. Non-calcified plaque is attached to the proximal part of the left anterior descending coronary artery (red arrow) and its distal side. The non-calcified plaque is long in the long-axis direction, and myocardial ischemia caused by this plaque is suspected.

Case3:

A 69-year-old man had developed skin eruptions that had not healed on the head and genital area 2–3 years earlier, but was monitoring his condition while using topical drugs prescribed by a local physician. Recently, he developed pain in his fingers, and visited the rheumatology department in our hospital. Psoriasis was suspected, and he subsequently visited the dermatology department. On that visit, scaly erythema was observed only on the head and genital areas, and psoriasis was identified from biopsy specimens (Figure 3a, b). PASI score was 2, BMI was 22.15 kg/m2, and results of blood tests and blood pressure were normal. CT of the heart revealed 80% stenosis of the 4th segment of the right coronary artery (Figure 3c). As a result, coronary artery bypass grafting was performed.

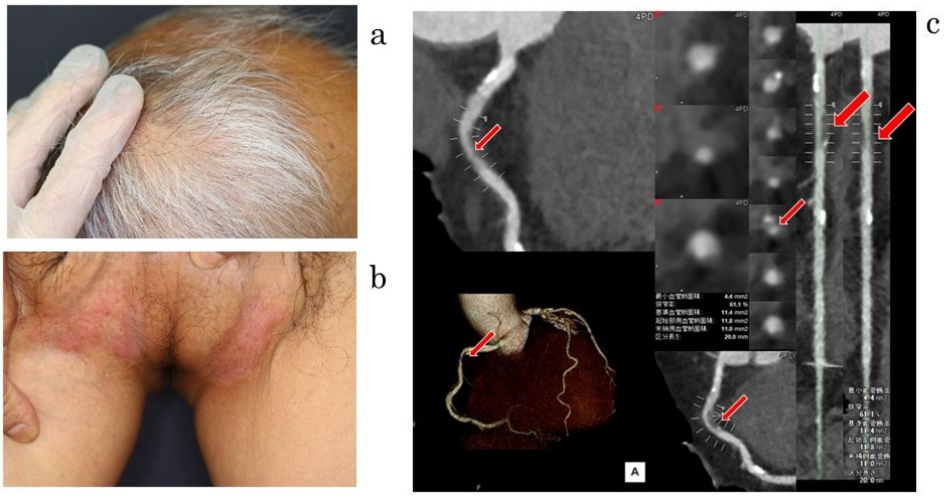

Figure S3. Mild case of psoriasis, Figure S3a, b: Erythema with scales is observed on the head (**a**) and both inguinal parts (**b**). Psoriasis was diagnosed by pathological examination of the inguinal

region. The patient had pain in both his fingers and his wrists. His PASI score was 2. **Figure S3c:** A non-calcified plaque (red arrow) was found proximal to the descending portion of the right coronary artery. The plaque is attached in the long axis direction and the CT value is low, suggesting that the plaque is fragile and unstable. BMI: body mass index, CCTA: coronary artery and cardiac computed tomography, CPR: curved plane reconstruction, PASI: psoriasis area and severity index, PV: psoriasis vulgaris, VR: volume-rendering.
